# An overview of the research progress on Mylabris: entomology, active ingredients, traditional use, pharmacology, clinical application, pharmacokinetics, toxicity and detoxification strategies

**DOI:** 10.1186/s13020-025-01257-0

**Published:** 2025-12-04

**Authors:** Qiyi Wang, Huan Zhang, Dingyang Lu, Wen Zhang, Sali Li, Hulang Ling, Xiaofei Li, Jianyong Zhang

**Affiliations:** 1 Medical College, Guizhou University, Guiyang, 550025 China; 2 Guizhou Provincial Key Laboratory of Innovation and Manufacturing for Pharmaceuticals, Zunyi Medical University, No.6 West Xuefu Road, Zunyi, 563000 China; 3 School of Pharmacy, Zunyi Medical University, Zunyi, 563000 China; 4 School of Basic Medicine, Zunyi Medical University, Zunyi, 563000 China; 5 State Key Laboratory of Discovery and Utilization of Functional Components in Traditional Chinese Medicine, Guizhou Medical University, Guiyang, 550014 China; 6 State Key Laboratory for Quality Ensurance and Sustainable Use of Dao-di Herbs, Beijing, 100700 China

**Keywords:** Mylabris, Entomology, Active ingredients and activity, Traditional and clinical uses, Toxicology

## Abstract

**Abstract:**

Mylabris, a traditional Chinese medicine (TCM), is derived from the dried forms of *Mylabris phalerata* Pallas or *Mylabris cichorii* Linnaeus. It was recorded in Shennong Bencaojing in Han Dynasty and used for the treatment of psoriasis, facial paralysis, amenorrhea, and carbuncle. As a key component in antitumor formulations, Mylabris contains numerous bioactive compounds, including organic acids, terpenoids, amino acids and their conjugates, metal complexes, cantharimide dimers and peptides and proteins. Traditionally, Mylabris has been employed in the treatment of malaria, suppurative infectious diseases, and lymph node tuberculosis. Pharmacological studies have demonstrated its antitumor, anti-inflammatory, leukocytosis-inducing, and immune function-enhancing activities, as well as its pest resistance and skin blistering effects. Clinical prescriptions containing Mylabris have been used in the treatment of cancer and skin diseases. However, strong penetration and rapid absorption in all tissues contribute to multi-organ toxicity on the liver, kidney, heart, nerves and reproduction and gastrointestinal systems. Therefore, traditional processing methods and targeted drug delivery systems have been designed for increasing efficacy and decreasing toxicity. Here, we provide a comprehensive overview of Mylabris in terms of entomology, active ingredients, traditional use, pharmacology, clinical application, pharmacokinetics, toxicity, and detoxification strategies to provide a rational application in the future.

**Graphical Abstract:**

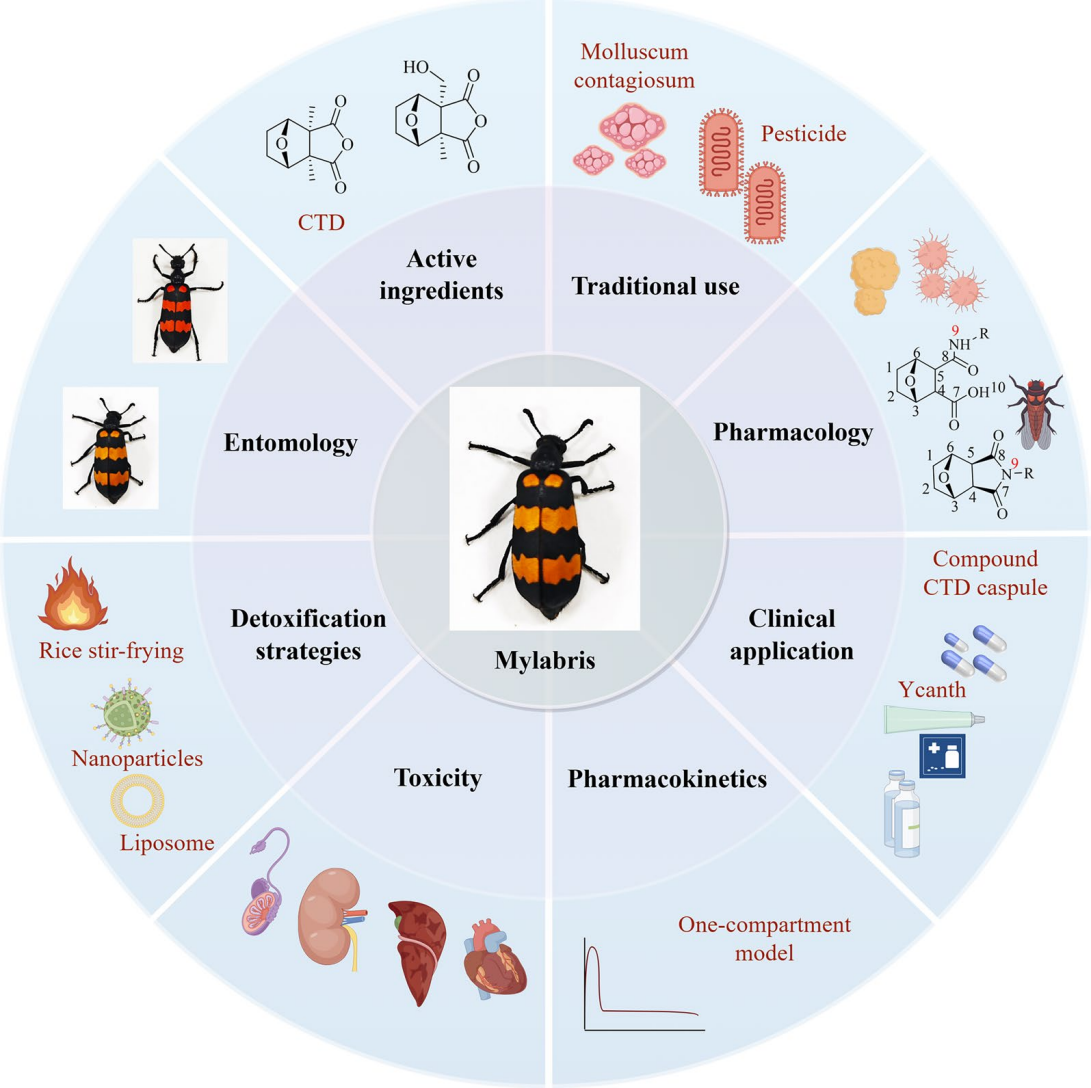

## Introduction

Mylabris, commonly known as “Spanish fly”, is the dried form of the Chinese blister beetle and is included in the 2020 edition of the Chinese Pharmacopoeia [1]. As a traditional Chinese medicine (TCM), Mylabris can be traced back more than 2000 years and was first recorded in the Shennong Bencaojing (Shennongs Classic of Materia Medica) in the Han Dynasty [2]. It also has a long medical history as a diuretic and aphrodisiac in Europe, documented in the ancient Greek medical monograph Materia Medica by Pedanios Dioskorides (50–100 AD) [3]. While traditionally prescribed for conditions like psoriasis, facial paralysis, amenorrhea, carbuncles, and warts via oral or topical routes, modern applications predominantly focus on oncology and dermatology. Currently, cantharidin (CTD), cantharidic acid (CA), calcium cantharidinate, sodium cantharidinate (SC) and other active ingredients have been isolated and identified from Mylabris. Pharmacological studies have confirmed that Mylabris has anticancer, immunomodulatory, and leukopoietic activities [3]. Clinically, prescriptions containing Mylabris like “Compound CTD Capsule,” “Delisheng Injection”, and “CTD Cream” are widely used in the treatment of liver and bladder cancers, leukemia and dermatological disorders. However, toxicity concerns persist. Following oral administration, CTD rapidly distributes across tissues under a one-compartment model, with pronounced accumulation in the liver and kidneys, which contributes to gastrointestinal, renal, and liver damage [4]. To mitigate these risks, traditional processing methods of Mylabris included net processing, rice stir-frying, and boiling method for reducing toxicity [5, 6]. And now, targeted drug delivery systems encapsulating CTD into liposomes and micelles can improve the efficacy and safety of Mylabris.

Although Mylabris has been employed for thousands of years, there is still insufficient research on its active compounds, metabolism and pharmacological and toxic mechanisms. This review offers a more comprehensive overview than other studies, covering aspects of entomology, active ingredients, traditional use, pharmacology, clinical application, pharmacokinetics, toxicity, and detoxification strategies. The review further highlights future development prospects for Mylabris, which will facilitate a more informed investigation and safe and effective clinical application.

## Entomology

Mylabris belongs to the phylum Arthropoda, class Insecta, order Coleoptera, and family Meloidae, with significant morphological characteristics, and is mainly distributed in Henan, Guangxi, Anhui, Jiangsu, Hunan, Guizhou, and Fujian provinces in China, as well as Myanmar [7, 8]. Mylabris is usually oblong in shape, with a relatively flat body and a special odor and luster. The head is triangular or round, and the dorsal elytra are black with dense dots and brown or yellow stripes [9]. The 2020 edition of the Chinese Pharmacopoeia specifies it as *Mylabris phalerata* Pallas or *Mylabris cichorii* Linnaeus and shows distinct morphological characteristics macroscopically and microscopically [1].

**Fig. 1:**
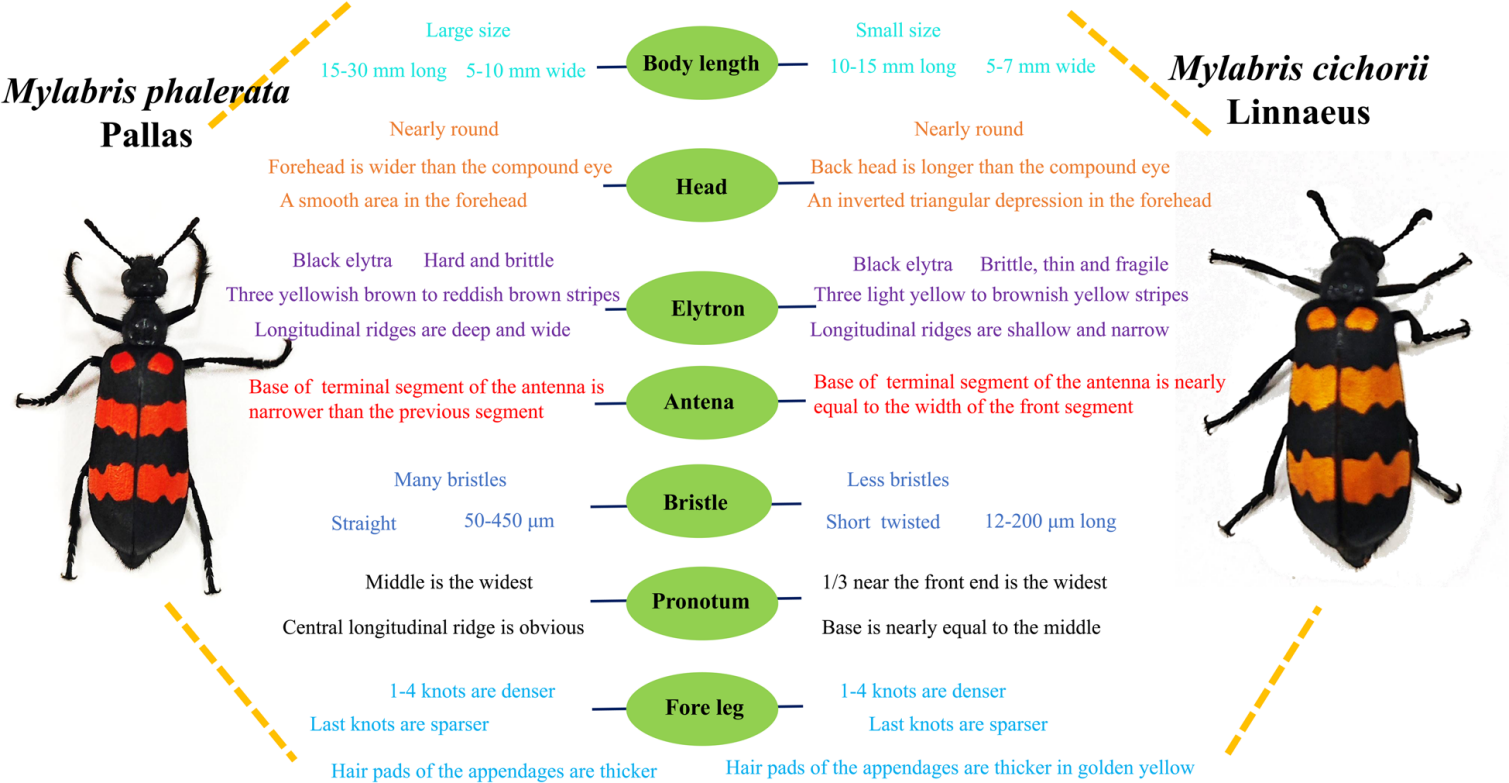
The distinctive characteristics of *Mylabris phalerata* Pallas and *Mylabris cichorii* Linnaeus

*Mylabris phalerata* Pallas is large in size. The head region is subcircular, 1.5–3.0 cm in length and 0.5–1.0 cm in width. The elytra are black, adorned with three stripes that vary from yellowish-brown to reddish-brown, hard and brittle, and the ventral surface has distinct, deep and broad longitudinal ridges. The morphology of the antennal is characterized by a terminal segment base being significantly narrower compared to the anterior segment. The dorsal surface is covered with numerous straight bristles, 50–450 μm in length, widest in the central area and with a prominent central longitudinal ridge. The appendages are covered with thicker hair pads, with denser knots at positions 1–4 and sparser distribution at the terminal knots (Fig. 1) [10, 11].

Conversely, *Mylabris cichorii* Linnaeus is small in size. The head region is subcircular, 1.0–1.5 cm in length and 0.5–0.7 cm in width. The elytra are black, decorated with three stripes varying from light yellow to brownish-yellow, brittle and thin, and break easily, with shallow and narrow longitudinal ridges on the ventral surface. The width of the base of terminal segment of antennae is approximately equal to that of anterior segment. The bristles are fewer, shorter, and occasionally twisted, 12–200 μm in length. Widest at the anterior end and almost equal to the middle at the base. The hair pads of appendages are thick in golden yellow, dense at positions 1–4, and sparse in the final section [10, 11]. The distinctive characteristics of *Mylabris phalerata* Pallas and *Mylabris cichorii* Linnaeus were shown in Fig. 1, contributing to their taxonomic identification and understanding of their biodiversity.

## Active ingredients

**Fig. 2:**
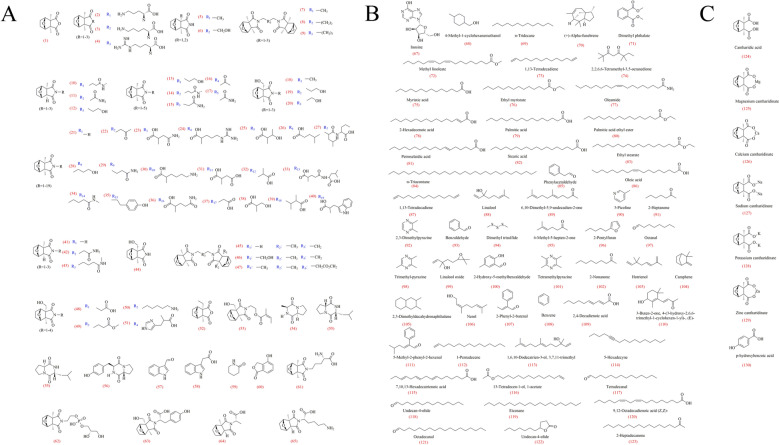
Active compounds in Mylabris. **A**: Sesquiterpenoids and their analogs, **B**: Volatile components, **C**: Other compounds

Mylabris contains numerous active ingredients, including organic acids, terpenoids, amino acids and their conjugates, metal complexes, cantharimide dimers and proteins (Fig. 2), which collectively provide the necessary energy for the survival and physiological function of Mylabris as well as for its structural stability.

### Sesquiterpenoids and their analogs

CTD (C₁₀H₁₂O₄) (1), a terpenoid found in Mylabris, was first isolated by Robiquet in 1810, and is the main active compound with significant antitumor activity [12]. In recent years, additional compounds have been progressively identified, demonstrating enhanced efficacy and reduced toxicity [13]. For instance, Takafumi discovered three novel amino acid conjugates through nucleophilic substitution at the anhydride oxygen, including L-lysine-, L-ornithine-, and L-arginine-modified analogs showing potent inhibition against protein phosphatases 1 (PP1) and 2A (PP2A) (2–4) [14]. Takafumie et al. further found three cantharimide dimers (consisting of two units of cantharimide combined with a tri-, tetra-, or penta-methylene group), as well as two cantharimides in Mylabris (5–9) [15]. Zeng et al. isolated 11 new monoterpenoids from Mylabis, including three 1-methyl cantharimide-type derivatives, five 1,2-dimethylcantharimide-type derivatives, and three 1-hydroxymethyl-2-methyl cantharimide-type derivatives, which were potent inhibitors of HBV viruses (10–20) [16]. In addition, Zeng identified 33 CTD analogs from the trichloromethane extract of Mylabris using ultra-performance liquid chromatography-quadrupole time-of-flight-tandem mass spectrometry (UPLC-Q-TOF). These analogs comprise 14 canthaminomides, 11 cantharimides, three palasoninimides, and five dicantharimides (7,8,13,15,18,19,21–47) [17]. Li et al. isolated five novel monoterpenoids from Mylabris, including three 1-hydroxymethyl-2-methyl cantharimide-type derivatives and two 1,2-dimethyl cantharimide-type derivatives, which demonstrated inhibition of renal fibrosis in vitro by suppressing transforming growth factor β1-induced expression of fibronectin and type I collagen in NRK-52e cells at a concentration of 40 μM (48–52) [18]. Zeng et al. isolated 9 compounds from Mylabris based on high-performance liquid chromatography and column chromatography over middle chromatogram isolated gel, including 5’-[(1R, 2R, 3S, 6R)-1-hydroxymethyl-2-methyl-3,6-epoxycyclohexane-1,2-dicarboximide]-ethyl-2’-methyl-2’-butenoate, cyclo-(L-Pro-L-Ala), cyclo-(R-Pro-R-Leu), cyclo-(S-Pro-R-Leu), cyclo-(D-Pro-L-Tyr), indole-3-aldehyde, 3-indoleacetic acid, valerolactam, and 4-hydroxyphthalid, which show lower antitumor activity than CTD (53–61) [19]. Recently, Li et al. identified five novel monoterpenoids, comprising two derivatives of the 1,2-dimethyl cantharimide type, one derivative of the 1-hydroxymethyl-2-methyl cantharimide type, and two derivatives of the 1-methyl cantharimide type, which may have potential therapeutic applications in the treatment of neuroinflammatory diseases (62–66) (Fig. 2A) [20].

### Volatile components

In addition to the sesquiterpenoids and their analogs, Pei et al. identified inosine (67), 4-methyl-1-cyclohexanemethanol (68), N-tridecane (69), ( +)-alpha-fullerene (70), dimethyl phthalate (71), methyl linoleate (72), and 1,13-tetradecadiene (73) in Mylabris through solid-phase microextraction (SPME) and gas chromatography-mass spectrometry (GC–MS) analysis (Fig. 2B) [21]. Li et al. detected 2,2,6,6-tetramethyl-3,5-octanedione (74), myristic acid (75), ethyl myristate (76), oleamide (77), 2-hexadecenoic acid (78), palmitic acid (79), palmitic acid ethyl ester (80), petroselaidic acid (81), stearic acid (82), ethyl stearate (83), and N-triacontane (84) across seven different provinces in China using GC–MS (Fig. 2B) [22]. Furthermore, Li et al. identified 41 compounds in *Mylabris phalerata* Pallas, with phenylacetaldehyde (4.07%) (85), oleic acid (3.21%) (86), 1,13-tetradecadiene (2.22%) (87), linalool (1.70%) (88), and 6,10-dimethyl-5,9-undecadien-2-one (1.64%) (89) present at higher concentrations than other compounds (73,82,90–123) [23].

### Peptides and proteins

Peptides and proteins are also important active ingredients of Mylabris. Liao et al. have extracted peptides with molecular weights between 1 and 5 kDa from Mylabris utilizing biomimetic enzymatic hydrolysis, which had notable antitumor activity without exhibiting immunosuppressive effects [24]. Wang et al. isolated a fibrinolytic protein from Mylabris with a molecular weight of 95.5 kDa, and showed that it effectively dissolved thrombi by activating plasminogen, without inducing hemolytic reactions [25].

### Other compounds

Li et al. identified numerous metal ions, such as calcium, magnesium, sodium, potassium, and zinc, in Mylabris. Furtherly, they employed acid hydrolysis and direct extraction methods to isolate total CTD and free CTD from eight wild Mylabris species, indicating the concentration of total CTD in Mylabris significantly exceeded that of free CTD, which suggested CTD may interact with hydrolysis products CA [26], to form magnesium cantharidinate, SC, calcium cantharidinate, potassium cantharidinate, zinc cantharidinate, and other compounds within Mylabris, which could inhibit the malignant proliferation ability of SMMC-7721 (124–129) [27–29]. Additionally, Liu et al. detected the presence of p-hydroxybenzoic acid in Mylabris, which expanded the range of chemical compounds of Mylabris (130) (Fig. 2C) [30].

The identification and investigation of these bioactive compounds offer a range of potential candidate molecules for the development of novel pharmaceuticals derived from Mylabris, thereby facilitating its further exploration and application.

## Traditional use

Mylabris has been medicinally utilized across civilizations for millennia. According to the records of the Shennong Bencaojing, Mylabris was characterized by a bitter and cold nature and was associated with the stomach, liver, and kidney meridians [2]. It has been traditionally employed in the treatment of malaria, suppurative infectious diseases, and lymph node tuberculosis. The classical Chinese medical literature has documented the use of Mylabris in removing necrotic tissue and eliminating scrofula. In Europe, the ancient Greek medical monograph “Materia Medica” documented that Mylabris was irritating and corrosive, employing it clinically for the topical removal of warts and cutaneous granulomas [3]. The ancient Greeks, Romans and Hippocrates used Mylabris as a diuretic, abortifacient, and aphrodisiac for the treatment of rabies, fevers, dropsy and many other systemic maladies [31, 32]. Additionally, Mylabris has therapeutic efficacy in addressing female gynecological disorders, various forms of tinea, sudden deafness, and rabies [3, 33]. The traditional use of Mylabris was summarized in Table 1. Beyond the main efficacy of TCM, different ethnic medicinal practices, such as Tibetan, Mongolian, Uygur, and others, have also utilized Mylabris for various disease conditions, including food accumulation, sand disease, vitiligo, eczema, mouth and eye deviation and throat infections [34, 35].

**Table 1:** The traditional use of Mylabris in China

Disease symptom	Using method	Source	References
Parasitic infection	Mylabris, peach peel, and euphorbia (1:2:2) were made into pills, with rice soup to serve	Handbook of Prescriptions for Emergency	[164]
Scrub typhus	Two Mylabris, one oral, the other burned to ash and applied to the affected area	Zheng Lei Ben Cao	[165]
Carbuncle	The Mylabris were crushed into powder, supplemented with garlic mud and water paste in the affected area	Ren Zhai Zhi Zhi Fang Theory	[166]
Psoriasis	After stir-frying, grind Mylabris into powder, it was mixed evenly with honey, and applied to the affected area	Compendium of Materia Medica	[167]
Melanocytic nevi	Fried Mylabris until light yellow, removed the rice, mixed garlic, and applied it to the affected area	Compendium of Materia Medica	[167]
Amenorrhea	Mylabris, frankincense, myrrh, catechu, croton, and onion were made into pills, wrapped with a cotton cloth and put into the vagina. Compatibility of peach kernel and rhubarb, taken orally	Ben Cao Yi Du	[33]
Deafness	Mixed the no-oil croton, Mylabris and musk with onion salivary and honey, rubbed into wheat grain size, wrapped with silk cotton and put into the ear	Yi Zong Jin Jian	[168]
Tuberculosis of lymph nodes	Fried rice flour and added mint powder, take orally	Compendium of Materia Medica	[167]
Bite of a rabid dog	Fried rice into powder, added oil to cold water and mix well, take orally	Essentials of Materia Medica	[169]
Bite of a rabid dog	After removing the wings, the Mylabris were burned yellow and mashed with toads, taken orally	Ben Cao Qiu Zhen	[170]

## Pharmacology

Mylabris has a longstanding history of utilization, with numerous researchers substantiating its efficacy, including combating cancers, reducing inflammations, elevating white blood cell counts, and enhancing immune function, as well as in pest control and the treatment of dermatological disorders, which indicates it has potential for clinical applications. The pharmacological activities were shown in Tables 2 and 3.

**Table 2:** The antitumour activity of Mylabris, CTD, and preparations containing Mylabris and CTD

Types of cancer	Administered preparations	Cell line/animal	Dosages	Times	Mechanism/results	References
Lung carcinoma	Delisheng Injection	PGCL3 cell	2, 5, 10, 25 μL/mL	24, 48, 72 h	Induced cell proliferation, adhesion, invasion, and migration	[38]
Osteosarcoma	CTD	U-2 OS, 143B, Saos-2, MG-63, MNNG cells / mice	1.25, 2.5, 5 μM / 2.5 mg/kg	24 h / 28 days	Inhibited proliferation and metastasis of osteosarcoma cells via down-regulating miR-214-3p and up-regulating DKK3 and reducing β-catenin nuclear translocation	[42]
Osteosarcoma	CTD	U-2 OS cell	6 μM	6, 12, 24, 48 h	Increased G2/M phase arrest and cell apoptosis	[43]
Oral cancer	CTD	SAS, CAL-27, SCC-4 cells	1, 3, 5, 10, 30 μM	1, 2, 4, 6, 16, 18, 24 h	Induced apoptosis in oral squamous cell carcinoma cells via the JNK-regulated mitochondria and ERS-related signaling pathways	[44]
Oral cancer	CTD	UMSCC1, UMSCC14A, UMSCC14B cells	10 μM	4, 8, 12, 24, 48 h	Induced ERS and unfolded protein response-dependent apoptosis	[45]
Hepatocellular carcinoma	CTD	HepG2.2.15, Hep3B cells	1, 2, 3, 4, 6, 9, 12 μM	24, 48 h	Inhibited the proliferation and decreased expression of CDK1 and CCNB1	[46]
Hepatocellular carcinoma	CTD	Mice	0.25, 0.5, 1 mg/kg	14 days	Regulated epithelial-mesenchymal transition progression, cell cycle pathways associated with EZH2/H3K27me3, immune responses and apoptosis	[48]
Hepatocellular carcinoma	CTD	Mice	0.25, 0.5, 1 mg/kg	14 days	Regulated autophagy or mitophagy, transcription factor-related transcriptional regulation, and fatty acid metabolism pathways	[49]
Hepatocellular carcinoma	CTD	HepG2, MHCC-97H, Hep3B, MHCC-97L, SMMC-7721, Huh-7 cells / mice	1.25, 2.5, 5 μM / 0.1, 0.2, 0.4 mg/kg	48 h / 18 days	Inhibited cell proliferation and induced cell apoptosis by targeting EphB4 and inhibited JAK2/STAT3 and PI3K/Akt signaling pathways	[47]
Hepatocellular carcinoma	CTD	Hep 3B cell	50 μM	10, 30, 60 min	Inhibited the mitochondrial energy system and the progression of cell cycle phases	[50]
Breast cancer	CTD	MCF-7, MDA-MB-231, SK-BR-3 cells	1, 10, 100 μM	24, 48, 72, 96 h	Increased cell apoptosis	[51]
Breast cancer	CTD	MDA-MB-231, MCF-7 cells / mice	0.1, 0.5, 1, 2 μM / 0.2, 0.5 mg/kg	24 h / 21 days	Inhibited the PKM2 nuclear translocation and broke GLUT1/PKM2 glycolytic loop and transformed aerobic glycolysis to oxidation	[52]
Breast cancer	CTD	MDA-MB-231 cell	2.5, 5, 10, 20 μM	24, 48, 72 h	Inhibited cell cycle progression at the G2/M phase, suppressed cell migration and invasion and inhibited the MAPK signaling pathway	[53]
Triple negative breast cancer	CTD	BT474, MDA-MB-468/231, T47D, MCF-7, 4T1 cells / mice	1.5, 3, 6 μM / 0.1, 0.2, 0.4 mg/kg	48 h / 12 days	Inhibited cell proliferation by regulating miR-607-mediated downregulation of EGFR and inhibited PI3K/AKT/mTOR and ERK/MAPK signaling pathway	[54]
Triple-negative breast cancer	CTD	MDA-MB-231 cell	5, 10, 15 μM	6 h	Reduced EGFR-mediated STAT3 and Akt signaling pathways, and reduced cell proliferation, and induced apoptosis	[55]
Bladder cancer	CTD	TSGH8301 cell	1.25, 2.5, 5, 7.5, 10 μM	6, 24, 48 h	Induced cell death by inducing DNA damage and inhibiting DNA repair	[56]
Bladder cancer	CTD	HT24, RT4 cells / mice	1, 3, 5, 10 μM / 0.5 mg/kg	16, 18, 24 h / 21 days	Induced cell apoptosis through activating calcium / PKC-regulated ERS pathway	[57]
Melanoma	CTD	A375.S2 cell	1, 2, 3, 4, 5 μM	12, 24, 48 h	Inhibited the migration and invasion of A375.S2 cells through suppressing the activity and expression of MMP-2 and MMP-9	[61]
Colorectal cancer	CTD	HCT116 cell	10, 20, 30 μM	48 h	Inhibited proliferation and migration of HCT116 cells and promoted apoptosis	[58]
Colorectal cancer	CTD	Colo 205 cell	5, 10, 20, 40 μM	24, 48, 72 h	Induced G2/M phase arrest and apoptosis in colo 205 cells through inhibition of CDK1 activity and Caspase-dependent signaling pathways	[59]
Gastric cancer	CTD	MGC803, BGC823 cells	2.5, 5, 10, 15, 20 μM	24, 48, 72 h	Inhibited PI3K/Akt signaling pathway by downregulating CCAT1 and suppressed cell growth and migration and invasion	[60]
Lung cancer	CTD	H460 cell	5, 7.5, 10, 15, 30 μM	6, 12, 24,48 h	Activated Caspase-8 by mediating death receptors and mitochondrial dysfunction by increasing Bax to Bcl-xL, releasing cytochrome c, apoptosis-inducing factor, and endonuclease G, and inducing apoptosis	[66]
Cervical and lung cancers	CTD	HeLa, A549, NCI-H460 cells	1, 2, 3, 4, 6, 9 μM	24 h	Inhibited migration and invasion of cells by inhibiting Exo70 transamidation	[62]
Prostate cancer	CTD	DU145, LNCaP cells	0.25, 0.5, 1 μM	18 h	Down-regulation of cellular FLICE‑like inhibitory protein and up-regulation of death receptor 5 promoted tumor necrosis factor (TNF)‑related apoptosis‑inducing ligand -induced apoptosis in prostate cancer cells by regulating autophagic flux	[65]
Skin cancer	CTD	A431 cell / mice	5, 10, 15, 20, 25 μM, / 0.2, 1 mg/kg	24, 48, 28 h / 29 days	Inhibited the G0/G1 arrest induced by cyclin E, CDK6, and cyclin D, and induced the apoptosis of A431 cells through Caspase and mitochondrial-dependent pathways	[67]
Cholangiocarcinoma	CTD	QBC939 cell	3, 6, 10, 20, 40 μM	12, 24, 48, 72 h	Inhibited PP2A activity and activated the IKKα/IκBα/NF-κB pathway	[68]
Pancreatic cancer	CTD	PANC-1, CFPAC-1 cells	0.1, 0.3, 1, 3, 10 μM	24 h	Induced cells to exit the G0/G1 phase and arrest in the G2/M phase, enhanced cytotoxicity and suppressed DNA damage repair gene expression through JNK, ERK, PKC, p38, and NF-κB pathways	[63]
Glioblastoma multiforme	CTD	A172, U87MG cells	10, 20,50 μM	24 h	Reduced cell proliferation by downregulating myeloid zinc-finger 1 and c-MYC	[64]
Promyeloid leukemia	CTD	HL-60 cell	25 M	24 h	Decreased expression of genes involved in DNA replication and increased expression of genes in modulating cytokine production	[69]
Hepatocellular carcinoma	CA	SK-Hep-1, Huh-7 cells	5, 10, 20, 40 μM	24 h	Induced apoptosis through a p38-mediated apoptotic pathway	[71]
Leukemic	CA	HL-60 AML cell	2.5, 5, 10, 20 μM	24 h	Induction of G2/M cell-cycle arrest and apoptosis	[72]
Nasopharyngeal carcinoma	CA	NPC-39, NPC-BM, HONE-1 cells	2.5, 5, 10 μM	24 h	Induced apoptosis through a p38-mediated apoptotic pathway	[70]
Osteosarcoma	SC	MG-63 cell	0.2, 1, 5 μM	12, 24, 48, 72 h	Inhibited phosphorylation of AKT, then decreased the expression of cyclin D1, CDK4 and CDK6, and induced MG-63 cell G0/G1 phase arrest	[76]
Hepatocellular carcinoma	SC	HepG2 cell	2, 5, 12.5 μM	6, 12, 24 h	Induced HepG2 cell apoptosis through LC3 autophagy pathway	[73]
Pancreatic cancer	SC	PANC-1, Aspc-1, BxPC-3 cells / mice	10, 20, 40 μM / 0.3 mg/kg	72 h / 21 days	Activated p53-Caspase-dependent apoptosis and inhibited the activation of the JAK2-STAT3 pathway	[74]
Breast cancer	SC	MCF-7, MDA-MB-231 cells / mice	4, 8, 16 μM / 0.3 mg/kg	24, 48, 72 h / 21 days	Inhibited PP5 activity maintains phosphorylation of p53 and induced apoptosis and G0/G1 cycle arrest	[75]
Breast cancer	SC	MCF-7 cell / mice	1, 2, 4, 8, 16, 32, 64 μM / 0.3 mg/kg	24, 48, 72 h / 21 days	Promoted cell apoptosis by inhibiting the PI3K/Akt/mTOR pathway and inducing autophagy	[77]
Cervical cancer	SC	Caski-1, ME180 cells / mice	50, 100 μM /100 mg/kg	72 h / 35 days	Inhibited the activation of PI3K/Akt pathway by targeting PTPN1 and promoted the sensitivity of cells	[78]

*CTD* Cantharidin, *DKK3* Dickkopf-3, *JNK* c-Jun N-terminal kinase, *ERS* Endoplasmic reticulum stress, *CDK1/4/6* Cyclin-dependent kinase 1/4/6, *EphB4* Eph receptor B4, *JAK2* Janus kinase 2, *STAT3* Signal transducer and activator of transcription 3, *PI3K* Phosphatidylinositol-4,5-bisphosphate 3-kinase; *Akt* Protein kinase B, *LC3* Microtubule-associated protein 1 light chain 3, *PKM2* Pyruvate kinase M2, *GLUT1* Glucose transporter 1, *MAPK* Mitogen-activated protein kinase, *EGFR* Epidermal growth factor receptor, *PKC* Protein kinase C, *MMP2/9* Matrix metalloproteinases 2/9, *CCAT1* Colon cancer associated transcript 1, *PP2A/5* Protein phosphatases 2A/5, *NF‑κB* Nuclear factor‑κB, *IKKα* NF‑κB kinase subunit α, *IκBα* NF‑κB inhibitor α, *ERK* Extracellular signal-regulated kinase, *PKC* Protein kinase C, *c-MYC* Myelocytomatosis viral oncogene homolog, *JAK2* Janus kinase 2, *STAT3* Signal transducer and activator of transcription 3, *PTPN1* Protein tyrosine phosphatase non-receptor type 1, *CA* Cantharidic acid, *SC* Sodium cantharidinate

**Table 3:** The multifunctional pharmacological activities of Mylabris, CTD, and preparations containing Mylabris and CTD

Pharmacological effects	Administered preparations	Biological models	Dosages	Times	Mechanism/results	References
Proliferation effect on skin	CTD	Mice	0.1%	0–72 h	Initiated regeneration by inducing acute injury, triggering two waves of synchronous proliferation of basal cells	[79]
Insecticidal activity	CTD	*Plutella xylostella*	0, 2.5, 10, 20 μg/ml	The eggs that emerged on the whole plant were recorded after 24 h and until adults died	Significantly affected the feeding behavior of *P. xylostella* and had an antifeedant action on third-instar larvae of *P.* xylostella	[83]
Insecticidal activity	CTD	House fly, *Musca domestica* L	0.25, 0.5, 1, 2, 4, 8, 16, 32 mg/L	72 h	CTD significantly suppressed the growth of the house fly population by affecting pupation rates, adult emergence, and prolonging the development cycle. The LC_50_ was 2.45 mg/kg	[84]
Insecticidal activity	CTD	*Plutella xylostella*	500 μg/ mL	Not provided	CTD showed significant larvicidal activity, with a larval mortality rate of 100%	[85]
Antibacterial activity	Aqueous extract of Mylabris	*Escherichia coli* 652654/ ATCC25922/ ATCC8739, *Klebsiella pneumoniae* 203/ ATCC13883, *Providencia rettgeri* 652655, *Pseudomonas aeruginosa* ATCC9027/ ATCC27853, *Salmonella abony* NCTC6017, *Salmonella enteritidis* ATCC13076, *Salmonella typhimurium* ATCC14028, *Serratia odorifera* 652411	Not provided	24 h	Inhibited 58.33% of the bacterial strains tested, and inhibitory activity was more remarkable against *Salmonella enteritidis* ATCC13076 (30 ± 0.0 mm), unlike *Escherichia coli* 652654 (10.0 ± 0.0 mm). In contrast, the extract had no inhibitory effect for *Escherichia coli* ATCC25922, the two strains of Klebsiella tested, *Pseudomonas aeruginosa* ATCC9027, and *Serratia odorifera* 652411	[86]
Antiparasitic activity	Dichloromethane extract of Mylabris	Nematode, fungi, nematodes, ticks, and protozoa	0.06, 0.12, 0.25, 0.5, 1 μg/μ L (Nematicidal); 0.1, 0.2, 0.4, 0.8 μg/μL (Antiprotozoal); 800 μg/mL (Fungicidal); 40 μg/μL (Ixodicidal)	24, 72 h	No bioactivity was observed in fungus and ticks, and exhibited dose-dependent nematicidal and trichomonicidal effects	[87]
Anti-inflammatory activity	CTD	The head kidney leucocytes of *Sparus aurata*	2.5, 5 μg/mL	24 h	Increased peroxidase activity and inhibited phagocytic ability	[88]
Positive inotropic effect	CTD	Mice, isolated human atria	10–100 μM (Mice); 10–300 μM (Isolated human atria)	15 min	Increased the absolute values of the rate of tension development and the rate of relaxation in the left atrial preparations through enhancing phosphorylation of phospholamban and Tnl	[89]
Increase in the force of contraction	CTD	Mice, isolated human atria	100 μM	30 min	Inhibited the activity of serine/threonine protein PP1 and PP2A and increased the force of contraction in human atrial preparations	[90]
Reversal of drug resistance	CTD	H9, HH cells / mice	0.39–25 mM / 0.5 mg/kg	48 h / 14 days	Overcoming vorinostat resistance to cutaneous T-cell lymphoma by blocking interleukin-2 receptor α-related signaling via reactive oxygen species (ROS) -dependent manner	[91]

*CTD* Cantharidin, *LC*_*50*_ Lethal dose, *ROS* Reactive oxygen species, *PP2A/1* Protein phosphatases 2A/1

### Antitumor activity

#### Mylabris

Mylabris exhibits antitumor properties, however, there is a paucity of research specifically investigating the antitumor effects of Mylabris itself. Mylabris was cooked with eggs, and then the eggs were consumed after discarding the Mylabris, as a traditional remedy for liver cancer and other cancers of the digestive system [36]. Now, prescriptions containing Mylabris, including “Compound CTD Capsule”, “Delisheng Injection” and “Ganning Tablets” are used for the treatment of liver cancer. The underlying antitumor mechanisms of Mylabris involve the reduction of cellular proliferation, promotion of apoptosis and cell cycle arrest, and attenuation of chemotactic and inflammatory responses [37–39]. In addition, a study indicated that Mylabris exerted a therapeutic effect on leukemia by triggering DNA damage, inducing apoptosis, as well as inhibiting the growth and proliferation of tumor cells through regulation of tumor protein 53 (TP53) and phosphatase and tensin homolog and p53 signaling pathway based on bioinformatics and systematic pharmacology [40].

#### CTD

**Fig. 3:**
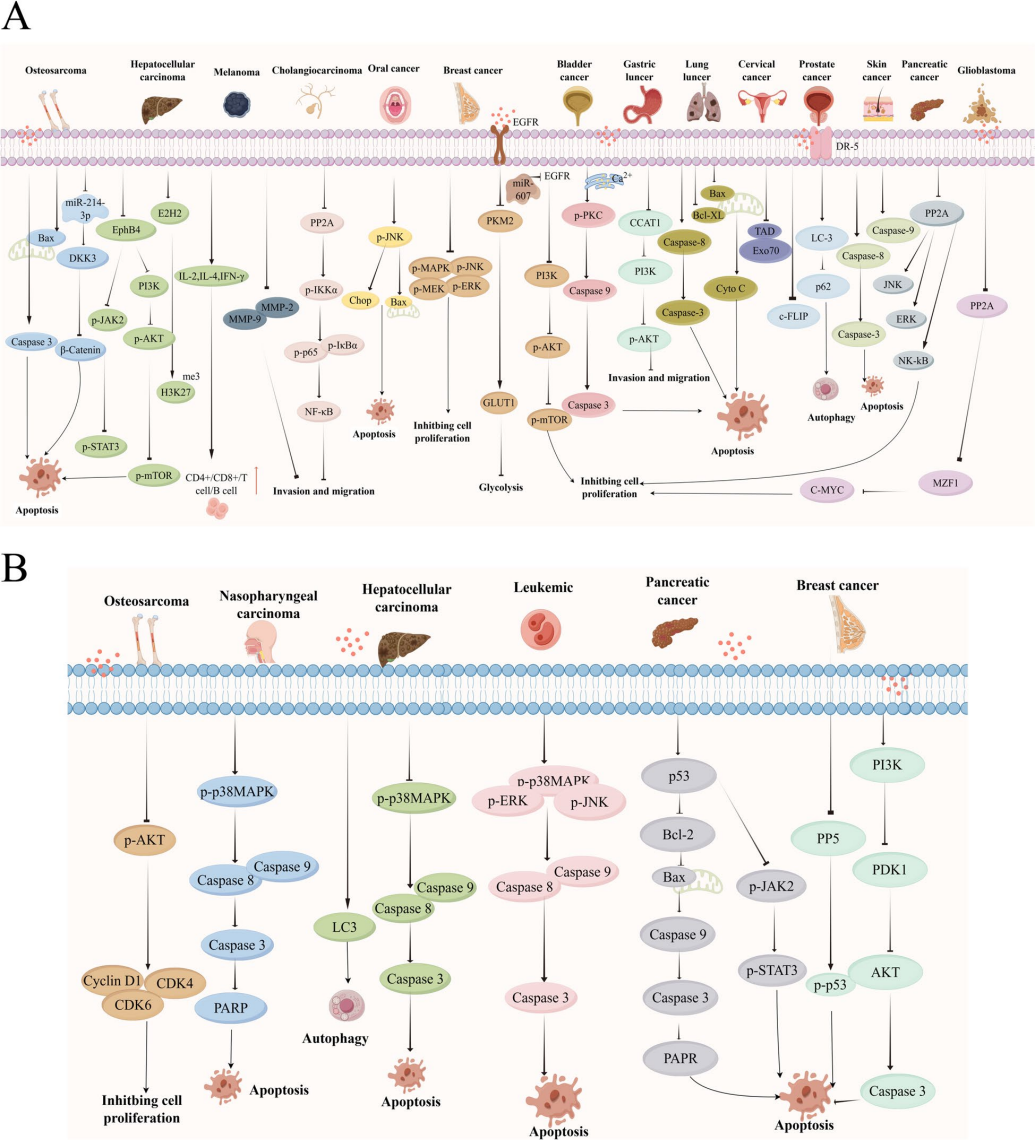
The antitumor mechanisms of Mylabris. **A**: CTD, **B**: Other active ingredients. (*CTD* Cantharidin, *DKK3* Dickkopf-3, *EphB4* Eph receptor B4, *JAK2* Janus kinase 2, *STAT3* Signal transducer and activator of transcription 3, *PI3K* Phosphatidylinositol-4,5-bisphosphate 3-kinase, *Akt* Protein kinase B, *MMP2/9* Matrix metalloproteinases 2 and 9, *PP2A/5* Protein phosphatases 2A/5, *NF‑κB* Nuclear factor‑κB, *IKKα* NF‑κB kinase subunit α, *IκBα* NF‑κB inhibitor α, *JNK* c-Jun N-terminal kinase, *CHOP* Enhancer-binding protein homologous protein, *MAPK* Mitogen-activated protein kinase, *ERK* Extracellular signal-regulated kinase, *PKM2* Pyruvate kinase M2, *GLUT1* Glucose transporter 1, *PKC* Protein kinase C, *CCAT1* Colon cancer associated transcript 1, *C-MYC* Myelocytomatosis viral oncogene homolog *CDK1/4/6* Cyclin-dependent kinase 1/4/6, *PARP* Poly ADP-ribose polymerase, *LC3* Microtubule-associated protein 1 light chain 3)

Modern pharmacological research has demonstrated CTD possesses antitumor properties, effectively inhibiting osteosarcoma, hepatocellular carcinoma, oral carcinoma, colorectal carcinoma, cervical carcinoma, lung carcinoma, gastric carcinoma, glioblastoma, breast carcinoma, cholangiocarcinoma, and leukemia. The underlying mechanisms involved the suppression of tumor cell proliferation, metastasis, and invasion, induction of DNA damage, modulation of immune responses, regulation of apoptotic pathways, and inhibition of glycolysis (Fig. 3A) [41].

For instance, in osteosarcoma, CTD downregulated miR-214-3p and upregulated dickkopf-3 (DKK3), leading to reduced nuclear translocation of β-catenin, thereby inhibiting cell proliferation and metastasis while enhancing apoptosis [42, 43]. In oral cancer, CTD decreased cell viability and activated apoptosis through activating c-Jun N-terminal kinase (JNK)-regulated mitochondrial and endoplasmic reticulum stress (ERS) signaling pathways [44, 45]. In hepatocellular carcinoma, CTD impeded cell proliferation and induce apoptosis by targeting the Eph receptor B4 (EphB4), as well as inhibiting the janus kinase 2 (JAK2)/signal transducer and activator of signal transducer and activator of transcription 3 (STAT3) and phosphatidylinositol-4,5-bisphosphate 3-kinase (PI3K)/protein kinase B (Akt) pathways [46, 47], and CTD regulated EZH2/H3K27 methylation pathway improved immunity [48, 49]. It also suppressed mitochondrial energy production and inhibited the progression of all phases of the Hep 3B cell cycle [50]. In breast cancer, CTD inhibited the nuclear translocation of pyruvate kinase M2 (PKM2), thereby triggering metabolic reprogramming and suppressing the activation of the mitogen-activated protein kinase (MAPK) pathway, which in turn impeded cellular proliferation, migration, and invasion [51–53]. Additionally, CTD upregulated miR-607, leading to the downregulation of epidermal growth factor receptor (EGFR) expression, and inhibition of the downstream PI3K/AKT/mTOR and extracellular signal-regulated kinase (ERK)/MAPK signaling [54, 55]. In bladder cancer, CTD induced DNA damage by activating the protein kinase C (PKC)-regulated ERS-dependent pathway [56, 57]. In colorectal cancer, CTD inhibited cyclin-dependent kinase 1 (CDK1) and promoted apoptosis [58, 59], while also suppressing the migration and invasion of gastric cancer cells by inhibiting the PI3K/Akt signaling pathway via colon cancer-associated transcript 1 (CCAT1) [60]. Additionally, CTD inhibited the expression of matrix metalloproteinases 2 and 9 (MMP2/9), interfered with the transamidation of Exo70, modulated PP2A levels, regulated autophagy, induce apoptosis and repair DNA repair for the treatment of melanoma [61], cervical cancer [62], pancreatic cancer [63], glioblastoma [64], prostate cancer [65], lung cancer [66], skin cancer [67], cholangiocarcinoma [68] and promyelocytic leukemia [69].

#### Other active ingredients

Some other active ingredients of Mylabris, such as CA and SC, have demonstrated promising antitumor activities. CA is the hydrolysis product of CTD and has been shown to induce apoptosis and inhibit cell migration and invasion in various cancers, including nasopharyngeal carcinoma, hepatocellular carcinoma, and leukemia [70–72]. In contrast to CTD, the anti-hepatocellular carcinoma and nasopharyngeal carcinoma effects of CA were primarily mediated through the p38-mediated apoptotic and microtubule-associated protein 1 light chain 3 (LC3) autophagy pathways [70, 71, 73]. In leukemia, CA induced apoptotic cell death by activating Caspase-8/9/3 signaling pathways and promoting polymerase cleavage through the activation of JNK1/2 in HL-60 cells. Additionally, SC exhibited anticancer properties in osteosarcoma, hepatocellular carcinoma, breast cancer and pancreatic cancer, as illustrated in Fig. 3B [73–77]. Mechanistically, SC suppressed the proliferation of human osteosarcoma MG-63 cells and induced cell cycle arrest by suppressing PI3K/AKT activation, thereby inhibiting the PI3K/Akt/mTOR signaling pathways and modulating energy metabolism through p53-dependent pathways to promote apoptosis in breast cancer cells [75–77]. Furthermore, SC targeted protein tyrosine phosphatase non-receptor type 1 (PTPN1) and inhibited the activation of the PI3K/AKT signaling pathway, presenting a potential treatment strategy for cervical cancer [78].

These findings highlight the diverse therapeutic potential of Mylabris in antitumor activity, underscoring its ability to engage multiple signaling pathways and cellular processes that are critical in the inhibition of cancer progression.

### Treatment of skin diseases

Mylabris acts as a vesicant agent, inducing the formation of external blisters upon dermal contact, and was employed in the treatment of warts, fungal infections, and neurodermatitis [3], but there were no modern pharmacological studies on it. Additionally, O. P. F. Clausen observed that CTD was applied to the backs of hairless mice, acting as a potent skin irritant capable of inducing regenerative proliferation of the skin [79].

### Insecticidal activity

Mylabris is not directly used for pest control. CTD is an inhibitor of protein serine/threonine phosphatases, which has been utilized as an innovative natural pesticide for managing Lepidoptera pests [80–82]. For example, Li et al. reported that CTD inhibited the oviposition of *Plutella xylostella* by 49.37% to 58.24%, and its repellent and antifeedant effects increased with higher CTD concentrations [83]. Hassan et al. observed CTD significantly reduced the population growth of house flies by affecting the pupation rate, adult emergence, and extending the developmental period, with a median lethal dose (LC_50_) value of 2.45 mg/kg [84]. Furthermore, Wen et al. synthesized two series of CTD analogs incorporating alkyl and aryl groups at the 9-position, which revealed that the structural form of CTD (whether cyclic or ring-opened) and its susceptibility to hydrolysis were critical factors influencing its efficacy against pre-third-instar *Plutella xylostella*. They further demonstrated that optimizing insecticidal activity necessitated a precise combination of both aliphatic amide and aromatic amide moieties [85].

### Antibacterial and antiparasitic activities

Mylabris contains defensins and toxins demonstrating antibacterial and antigen-clearing properties. Mamadou et al. reported that an aqueous extract of Mylabris inhibited 58.33% of the tested Gram-negative bacterial strains, with a particularly notable effect against *Salmonella enteritidis* ATCC13076 [86]. Additionally, Marta et al. indicated that a dichloromethane extract of Mylabris exhibited dose-dependent nematicidal and trichomonicidal effects, which could be attributed to the active components CTD and ethyl oleate [87].

### Other activities

Beyond the above activities, as an inhibitor of PP1/PP2A, CTD has been shown to enhance peroxidase activity in the head kidney leukocytes of gilthead seabream (*Sparus aurata*) and to exhibit anti-inflammatory properties. Furthermore, CTD increased the contractile force and reduced the relaxation time in human ventricular preparations, while also reversing drug resistance [88–91].

In summary, Mylabris and its active ingredients exhibit significant antitumor activity by inhibiting tumor cell proliferation, migration, invasion and apoptosis by regulating multiple signaling pathways and cell cycle pathways. Additionally, Mylabris has exhibited efficacy in the treatment of skin diseases, as well as insecticidal, antibacterial, and antiparasitic activities. These findings provide a robust scientific foundation for the potential clinical applications and drug development of Mylabris in the future.

## Clinical application

Mylabris and its preparations, along with their active compounds, exhibit significant antitumor and dermatological activities clinically [37, 92, 93]. The preparations were summarized in Table 4. The “Compound CTD Capsule” achieved 80% efficacy in stages III and IV nasopharyngeal carcinoma patients after one month of treatment, and with a superior safety profile and enhanced patient tolerance comparable to conventional chemotherapy [94]. Furthermore, the “Compound CTD Capsule” could reduce alpha-fetoprotein (AFP) levels and improve survival rates in patients with advanced primary liver cancer [37]. However, adverse reactions may occur in clinical applications, including elevated total bilirubin and serum creatinine levels, necessitating rigorous monitoring of hepatic and renal function during treatment [95]. “The Leech Mylabris Decoction” significantly improved symptoms of benign prostatic hyperplasia, reducing prostate volume by more than 20% with only mild gastrointestinal adverse effects after two months of treatment, yielding an overall efficacy rate of 93.33% [96]. In oncology, “Delisheng Injection” enhanced immune function in stage II-III nasopharyngeal carcinoma patients [97], while combined therapy with “SC Injection” reduced tumor volume and serum AFP levels in advanced hepatocellular carcinoma after 6–12 weeks of treatment [98, 99]. However, due to potential nephrotoxicity and urinary system complications, close renal function monitoring is mandatory, and therapy should be discontinued upon signs of organ damage [100]. Additionally, external preparations like “Mylabris Mustard Ointment” had an effective rate of 100% in the treatment of tennis elbow management [101], while “Croton Mylabris Ointment” achieved a 94.3% efficacy rate in treating peripheral facial paralysis [102]. Some Mylabris formulations have demonstrated clinical efficacy in dermatologic applications. “CTD Cream” showed superior performance compared to CO₂ laser and cryotherapy in the treatment of verruca plantaris, but it is crucial to ensure that the daily dosage does not exceed 3 g to prevent the risk of skin necrosis [103]. In pediatric molluscum contagiosum, topical 0.7% CTD (VP-102) demonstrates high effectiveness despite transient adverse effects including vesicles, pain, pruritus, erythema, and scabbing [104–106]. Case reports also further corroborate its efficacy for resistant plantar warts and genital warts [107, 108]. While pharmacological studies have demonstrated that Mylabris possesses notable antibacterial and antiparasitic properties, there remains a lack of direct clinical evidence substantiating its efficacy. Further research can develop Mylabris as a more effective antimicrobial agent and explore its promising pharmacological activity in treating parasitic infections.

**Table 4:** Preparations containing Mylabris or its related active ingredients

Preparations	Countries	Drug dosage forms	Composition	Indications	Specification	Usage	Maximum clinical efficiency	References
Compound CTD capsule	China	Capsule	Mylabris, Panax ginseng, Astragali Radix, Acanthopanax senticosus, Sparganii Rhizoma, Scutellaria barbata, Curcuma zedoaria, Corni Fructus, Ligustri Lucidi Fructus, Bear bile powder, Licorice, etc	Primary liver cancer, lung cancer, rectal cancer, malignant lymphoma, gynecological malignant tumor	0.25 g/capsule	Oral	95.80%	[171, 172]
Delisheng injection	China	Injection	Mylabris, Red ginseng, Astragali Radix, Bufonis Venenum	Middle and advanced primary liver cancer with Qi deficiency and blood stasis syndrome	10 mL each	Intravenous drip	93.75%	[38, 173]
Ganning tablets	China	Tablet	Mylabris, Armillaria Pachyderma, Glutinous rice	Acute and chronic hepatitis, abnormal liver function, hepatitis B patients, prevention of hepatitis B cancer	Each tablet weighs 0.3 g	Oral	95.45%	[39]
SC and vitamin B6 injection	China	Injection	SC and Vitamin B6	Liver cancer, lung cancer, leukopenia, hepatitis, cirrhosis, hepatitis B virus carriers	0.05 mg/5 mL, 0.1 mg/10 mL	Intravenous drip	92.00%	[174, 175]
Mylabris mustard ointment	China	Cream	Mylabris, White mustard, Bone-seeking Wind	Tennis elbow management	Appropriate amount	Topical application	100.00%	[101]
Croton Mylabris ointment	China	Cream	Croton, Mylabris, Ginger	Peripheral facial paralysis	Appropriate amount	Topical application	94.30%	[102]
SC injection	China	Injection	SC	Primary liver cancer, esophageal cancer	0.1 mg/2 mL, 0.25 mg/ mL, 0.5 mg/10 mL	Intravenous drip	91.07%	[176, 177]
CTD cream	China	Cream	CTD	Genital warts	1 mg/4 g	Topical application	90.00%	[103, 178]
Ycanth (VP-102)	USA	Solution	CTD	Molluscum Contagiosum	0.7% W/V	Topical application	85.70%	[106]

*CTD* Cantharidin, *SC* Sodium cantharidinate

In summary, the wide application of prescriptions containing Mylabris in the clinic showed that Mylabris has potential and application prospects.

## Pharmacokinetics

Pharmacokinetics is crucial for understanding the metabolic regularity of drugs in vivo. Duan et al. demonstrated the aqueous extract of Mylabris was rapidly absorbed systemically in rats, reaching peak plasma concentrations within 0.17 h post-administration [4]. CTD showed a faster elimination rate and exhibited a one-compartment model pharmacokinetic profile in canine plasma with an elimination half-life of 0.69 ± 0.03 h, with an area under the curve of 204 ± 24 h·ng/mL based on gas chromatography-mass spectrometry [109]. In contrast, CA showed longer elimination kinetics with a half-life of 1.2 h and AUC of 158 h·ng/mL in rats [110].

Mylabris showed strong penetration in tissues, mainly in the kidney at low concentrations and in the spleen at high concentrations in rats [4]. Additionally, Zhang et al. indicated that the concentration of CTD peaked in the liver and kidneys at 72 h postmortem before subsequently declining. A significant correlation was observed between liver CTD concentration and postmortem interval (linear regression R^2^ = 0.863), while the correlation in the kidneys was weaker (R^2^ = 0.115), and liver CTD concentration could serve as a biomarker for postmortem interval estimation in rats [111].

In summary, investigation of the absorption, distribution, metabolism and excretion will provide insight into the in vivo behavior and mechanism of Mylabris.

## Toxicity

**Fig. 4:**
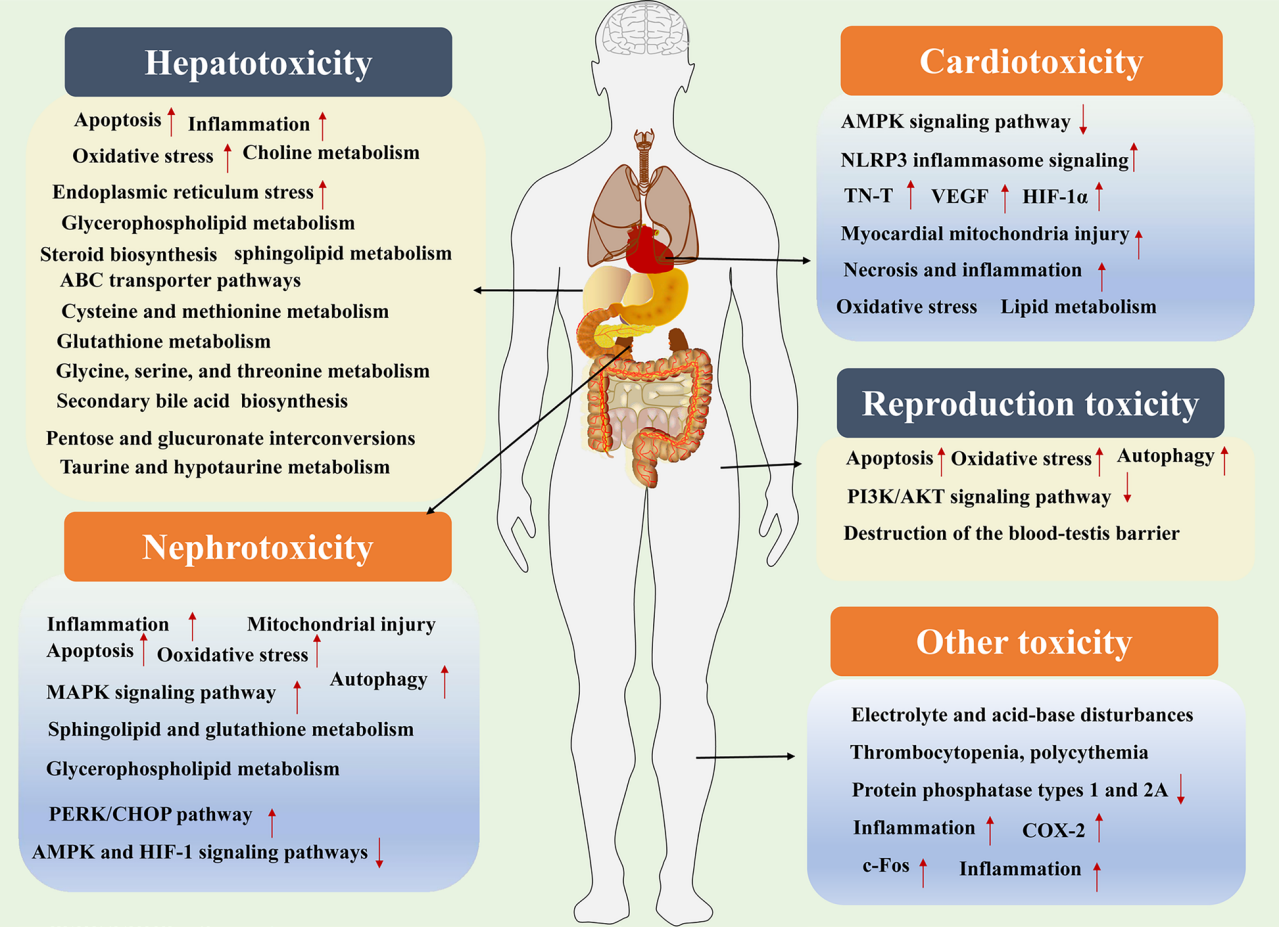
The toxicity mechanisms of Mylabris. (*MAPK* Mitogen-activated protein kinase, *PERK* Protein kinase RNA-like endoplasmic reticulum kinase, *CHOP* Enhancer-binding protein homologous protein, *AMPK* AMP-activated protein kinase; *HIF-1* Hypoxia-inducible factor-1, *NLRP3* Nucleotide-binding oligomerization domain, leucine-rich repeat and pyrin domain-containing 3, TN-T Troponin T, *VEGF* Vascular endothelial growth factor, *PI3K* Phosphatidylinositol-4,5-bisphosphate 3-kinase, *Akt* Protein kinase B, *COX-2* Cyclooxygenase-2)

Inexperienced use and vague dosage guidance in traditional Mylabris applications frequently lead to overdose and toxicity. European cases commonly involve CTD poisoning from ingestion as a sexual stimulant [112]. Clinical manifestations of external poisoning include blistering, erythema, and erosion, whereas oral poisoning is frequently associated with symptoms such as gastrointestinal burning, emesis, palpitations, chest constriction, coma, and hematuria. Mortality in most cases was attributed to acute renal failure, acute circulatory collapse, or multiple organ dysfunction syndrome [113]. CTD is identified as the primary toxic component of Mylabris, with numerous toxic effects documented in both humans and animals [114–116]. Research indicated that the acute median lethal dose (LD_50_) for intraperitoneal administration in mice was 1.71 mg/kg, while the lethal dose for humans was 30 mg [3]. Regardless of whether CTD is administered orally or topically, it is prone to inducing acute, critical, and severe toxic reactions. These reactions encompassed gastrointestinal, dermal, hepatic, and renal damage, accompanied by alterations in biochemical functions and pathological states. The underlying mechanisms of toxicity were associated with inflammation, apoptosis, and oxidative stress (Fig. 4).

### Hepatotoxicity

The hepatotoxicity of Mylabris was primarily characterized by single-cell necrosis, steatosis, increased white blood cell infiltration in the hepatic sinusoids, mitochondrial damage, elevated autophagic vacuoles, smooth endoplasmic reticulum proliferation, and nuclear chromatin condensation [117].

The hepatotoxicity of CTD was marked by jaundice, hepatosplenomegaly, and elevated levels of alanine aminotransferase (ALT) and aspartate aminotransferase (AST), hepatic steatocyte degeneration and necrosis, as well as liver inflammation [118]. The underlying mechanisms were primarily associated with oxidative stress, metabolic disturbances, inflammation, apoptosis, ERS, and autophagy. For instance, Liu et al. demonstrated that CTD treatment significantly increased the levels of ALT, AST, and lactate dehydrogenase (LDH) in LO2 cells, and CTD upregulated the mRNA expression of DNA methyltransferase 1 and nitric oxide synthase 1 [119]. They further found CTD activated autophagy and apoptosis via the ERS pathway in LO2 cells [120]. Furthermore, CTD exacerbated inflammatory responses through the activation of tumor necrosis factor-alpha and IκB kinase α signaling pathways, and induced apoptotic cascades by simultaneously upregulating Caspase-3 and Bax while downregulating the anti-apoptotic protein Bcl-2 in rats [121, 122]. Additionally, CTD could increase oxidative stress and ERS in mice [123, 124]. Metabolomic studies demonstrated that CTD could disrupt amino acid and glutathione metabolism in LO2 cells and mice, as well as interfere with bile acid biosynthesis and lipid metabolism in rats [119, 125–128]. Moreover, Wang et al. found that gender-specific dysregulation of glycerophospholipid metabolism, mediated by phosphatidate phosphatase 1, choline/ethanolamine phosphotransferase 1, and phosphatidylethanolamine N-methyltransferase, contributed to abnormal liver function and elevated triglyceride and total cholesterol levels in rats [129].

### Nephrotoxicity

The nephrotoxicity of Mylabris was mainly manifested as renal cell degeneration, necrosis, increased lysosomes, and the appearance of autophagic vacuoles in a dose-dependent manner [117].

The nephrotoxicity induced by CTD was primarily manifested as hematuria, proteinuria, dysuria, renal dysfunction, acute tubular necrosis and glomerular destruction [130]. The mechanisms mainly focused on oxidative stress, inflammation, autophagy, apoptosis and metabolic disorder. For instance, Liu et al. found CTD could increase uric acid and creatinine levels, active MAPK and adenosine monophosphate-activated protein kinase (AMPK) and hypoxia-inducible factor-1 (HIF-1) pathways and induce oxidative stress in mice [131]. Xiao et al. found CTD could induce apoptosis and oxidative damage in mice, mainly by disrupting sphingolipid and glutathione metabolism, increasing sphingosine and sphingomyelin levels, and decreasing glutathione levels [132]. He et al. found CTD could induce nephrotoxicity by inhibiting lipid metabolism [133], and activating the ERS-dependent protein kinase RNA-like endoplasmic reticulum kinase (PERK) /CCAAT/ enhancer-binding protein homologous protein (CHOP) pathway, triggering autophagy and apoptosis in HK-2 cells and rats [134]. Furtherly, Cheng et al. found that CTD-induced nephrotoxicity was related to pantothenate and CoA biosynthesis, and pyruvate metabolism, based on CatBoost Classifier prediction mode in rats [135].

### Cardiotoxicity

There is limited research on the cardiotoxic effects of Mylabris, and current pathological investigations have identified that Mylabris primarily induces cardiac congestion [117].

Research on cardiotoxicity has predominantly focused on CTD, and it can promote cardiomyocyte senescence and impair mitochondrial function. This impairment results in cardiac damage characterized by symptoms such as heart palpitations, reduced blood pressure, and electrocardiogram abnormalities, and pathologically by myocardial degeneration, necrosis and focal fusion. Shi et al. reported that CTD could decrease basal respiration, ATP levels, and spare respiratory capacity in H9c2 cardiomyocytes by inhibiting AMP-activated protein kinase and activating the leucine-rich repeat and pyrin domain-containing 3 (NLRP3) inflammasome [136]. Additionally, CTD could induce inflammation and myocardial interstitial hyperemia through activating oxidative stress [137]. CTD could also induce the cardiac muscle fibers, infiltration of inflammatory cells in the myocardium, and elevated serum troponin T (TN-T) levels by disrupting the extracellular matrix-receptor interaction pathway and fatty acid metabolism pathway in rats [138]. Further, Zhang et al. found TN-T, vascular endothelial growth factor, and hypoxia-inducible factor-1α in the serum of rats might be valuable molecular markers of CTD-induced cardiac injury [139].

### Reproductive toxicity

Mylabris could induce miscarriage, as recorded in the Shennong Bencaojing [2]. In a study, the aqueous extract of Mylabris was administered to female rats daily for 5 days before and 19 days following mating, resulting in a notable decrease in pregnancy rates and an increase in fetal malformations [140].

Moreover, CTD could induce testicular injuries and be characterized by reduced sperm count in spermatogenic tubules and vaginal bleeding. The underlying mechanisms involve inflammation, oxidative stress, cell infiltration, and necrosis [130, 141]. A case report indicated that ingestion of Mylabris is associated with suprapubic and flank pain, as well as vaginal bleeding [130]. Liu et al. observed that CTD could increase the testicular index and lead to congestion in the testicular space of mice through downregulation of Bcl-2, while upregulation of Bax and Caspase-3, and suppression of PI3K/AKT signaling pathway [141]. Furthermore, Xiao et al. demonstrated that exposure to CTD in mice could induce mitochondrial autophagy, cellular swelling in the testicular and, which was mediated by oxidative stress, enhanced autophagy levels, and the disruption of the blood-testis barrier [142].

### Other toxicities

Inappropriate administration of Mylabris and CTD also induced other toxicities, including hematolymphatic, gastrointestinal, bladder, dermal, and neurological toxicities [113]. Hematolymphatic injuries induced by Mylabris encompass lymphocytic necrosis, acute splenitis, and impaired immune regulation. Bladder toxicity was characterized by coagulative necrosis of mucosal epithelial cells in the urinary tract, accompanied by inflammatory cell infiltration, vascular congestion, and upregulation of cyclooxygenase-2 and the inflammation-related transcription factor c-fos in both RAW 264.7 and T24 cells, as well as in rats [143]. Gastrointestinal toxicity was accompanied by severe luminal distension, mucosal edema, and hyperemia of the mesenteric vasculature [117, 144]. Ocular damage presented with lacrimation, periocular edema, conjunctival congestion, and transient facial spasms [117, 144, 145]. Neurological symptoms include dizziness, headache, restlessness facial paralysis and psychiatric disturbances [146]. Dermal toxicities exhibited blisters on various body parts, such as the nape and buttocks [147].

In summary, Mylabris could cause multi-organ damage, including hepatotoxicity, nephrotoxicity, cardiotoxicity, reproductive, gastrointestinal, bladder and dermal toxicities, neurotoxicity and immunotoxicity, and a comprehensive understanding of the toxicological profile of Mylabris is essential for the development of strategies aimed at mitigating its adverse effects and ensuring the safety of therapeutic applications.

## Detoxification strategies

### Traditional processing

Due to the inherent toxicity of Mylabris, detoxification processes have been essential from historical to contemporary practices. Traditional detoxification primarily relies on thermal decomposition and chemical transformation. Historically, the methods involved the removal of cephalic, pedal, and alar structures followed by thermal processing with rice, vinegar, and oyster shells [148]. Currently, the methods involve stir-frying with rice until a yellowish-brown hue is achieved, followed by the removal of appendages, to facilitate toxin sublimation [148]. A study has shown that baking at 110 °C for 26–30 min produced CTD, formic acid, and fatty oil profiles comparable to those obtained through traditional stir-frying, establishing it as a viable alternative [149]. Furthermore, alkaline hydrolysis is a novel processing technique that uses low concentrations of sodium hydroxide and has been developed. This method facilitated the formation of SC from Mylabris, which not only reduced toxicity but also exhibited enhanced antitumor efficacy [6].

### New targeted delivery system

**Fig. 5:**
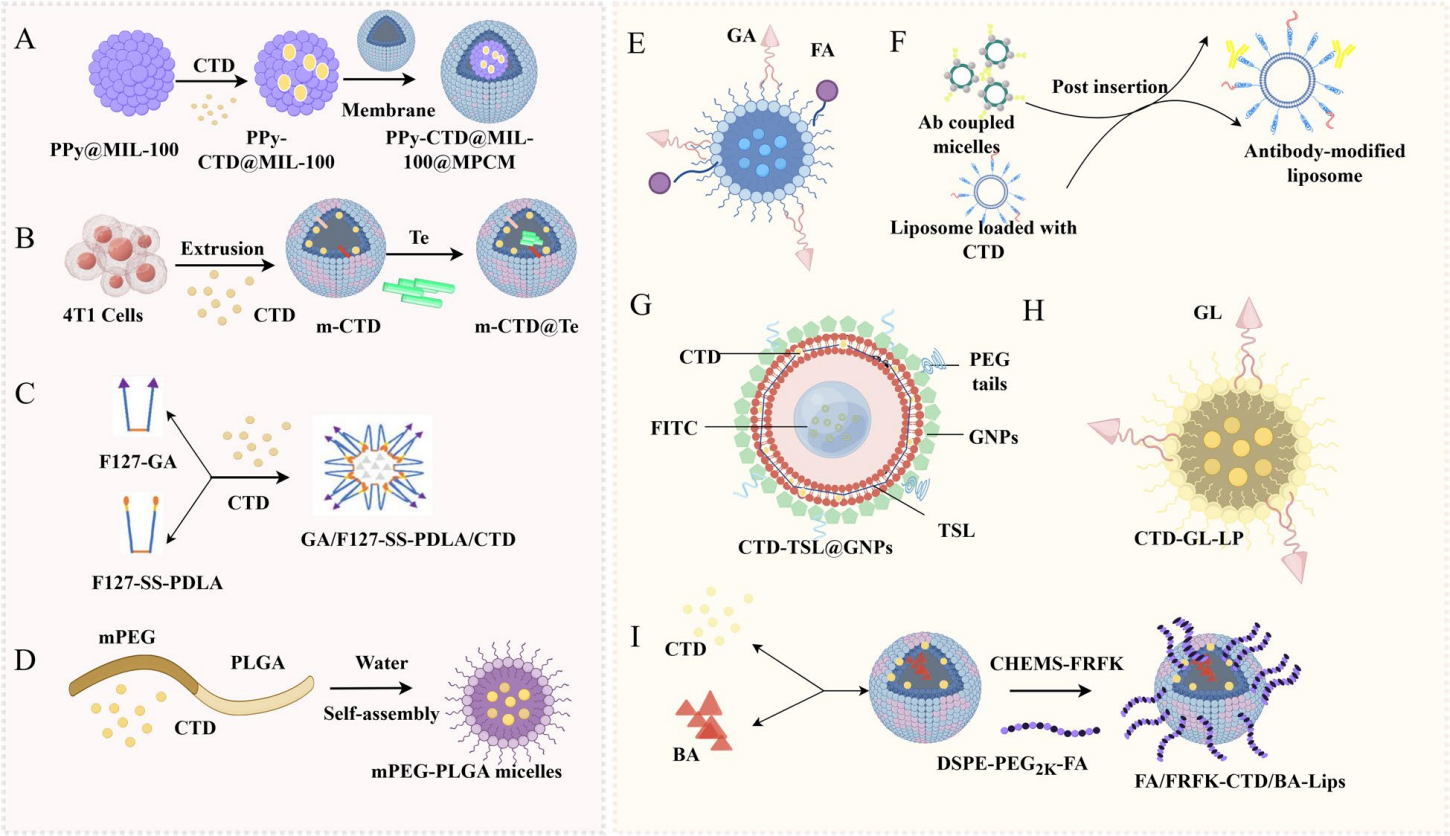
New targeted delivery systems of CTD. **A**: Schematic of metal–organic framework-based platforms integrating Fenton reaction and photothermal therapy. **B**: Schematic of biomimetic nano-drug delivery systems with tellurium and cancer cell membrane-derived nanocarriers. **C**: Schematic of redox-sensitive polymeric micelles modified with glycyrrhetinic acid. **D**: Schematic of mPEG-PLGA micelles. **E**: Schematic of GA and FA modified CTD loaded solid lipid nanoparticles. **F**: Schematic of carbonic anhydrase IX antibody and BR2 peptide modification dual-functional liposomes. **G**: Schematic of thermal-sensitive liposomes coated with gold nanoparticles. **H**: Schematic of GL-based liposome. **I**: Schematic of dual-modified CTD/baicalin co-loaded liposomes. (*CTD* Cantharidin, *GL* Glycyrrhizic acid, *GA* Glycyrrhetinic acid, *FA* Folate)

Researchers have explored targeted drug delivery systems to enhance the solubility, bioavailability, and efficacy of active compounds CTD and SC in Mylabris and validated them in in vitro and in vivo models. Various innovative tumor-targeted approaches have been developed, which have increased drug accumulation in tumor tissues and decreased accumulation in other organs. Such as metal–organic framework-based platforms integrating Fenton reaction and photothermal therapy, such as PPy-CTD@MIL-100@MPCM nanoparticles (PPy-CTD@MIL-100@MPCM) (Fig. 5A) [150], biomimetic nano-drug delivery systems with tellurium and cancer cell membrane-derived nanocarriers (m-CTD@Te) (Fig. 5B) [151], and redox-sensitive polymeric micelles modified with glycyrrhetinic acid(GA) (GA/F127-SS-PDLA/CTD micelles) (Fig. 5C) [152], hyaluronic acid-mPEG modified nanostructured lipid carriers(HA-mPEG-CTD-NLC) [153], mPEG-PLGA micelles[154] (Fig. 5D), GA and folate (FA) modified CTD loaded solid lipid nanoparticles (Fig. 5E) [155], and non-ionic surfactant vesicles [156] loaded with CTD, which inhibited the heat shock response in tumor cells and enhanced tumor cells apoptosis with high anticancer effect and low systemic toxicity.

Liposomes are effective carriers for targeted drug delivery, playing a key role in increasing the effectiveness and decreasing the toxicity of anticancer drugs. Research indicated that liposome-encapsulated CTD maintains its antitumor efficacy while offering the additional benefit of diminished systemic toxicity [157]. Taking advantage of this, a series of CTD-loaded liposomes have been developed to actively target tumor cells, including carbonic anhydrase IX antibody and BR2 peptide modification dual-functional liposomes (Fig. 5F) [158], 3-succinyl-30-stearyl glycyrrhetinic acid-modified liposomes (18-GA-Suc-CTD-Lip) [159], thermal-sensitive liposomes coated with gold nanoparticles(CTD-TSL@GNPs) (Fig. 4G) [160], L5 peptide modified Glypican-3 targeted liposome (L5-Lip/CTD) [161], glycyrrhizic acid-based liposome (CTD–GL–LP) (Fig. 5H) [162], and dual-modified CTD/baicalin co-loaded liposomes (FA/FRFK-CTD/BA-Lips) (Fig. 5I) were very useful for improving the effectiveness and safety of CTD [163].

While these in vivo targeting formulations significantly attenuate toxicity and enhance tumor-selective delivery, none have advanced to formal human clinical trials. Advancing clinical translational research on these strategies offers a promising opportunity to improve the clinical applicability and safety of Mylabris in cancer treatment.

## Conclusion and prospect

Mylabris has been used for thousands of years and is highly esteemed for its healing properties. However, it comes with substantial toxic side effects. Until now, many chemical compounds have been isolated and identified from Mylabris. Mylabris has exhibited a range of pharmacological activities, such as antitumor, anti-inflammatory, leukocytosis, immune function enhancement, pest resistance, and treatment of skin diseases through inhibition of tumor cell proliferation and metastasis, modulation of immune responses and apoptotic pathways through inhibiting PP1/PP2A, regulated PI3K/AKT/mTOR, ERK/MAPK, JAK2-STAT3, and other signaling pathways. Clinically, prescriptions such as “ Compound CTD Capsule,” “ Delisheng Injection,” and “ CTD Cream “ have demonstrated significant efficacy in anticancer and skin disease treatments. Despite the excellent pharmacological activities, especially the anticancer effect, there is substantial evidence supporting the strong hepatotoxicity, nephrotoxicity, cardiotoxicity, reproductive and dermal toxicities, with mechanisms involving glycerophospholipid metabolism, choline metabolism, MAPK, ABC transporter, and PI3K/AKT signaling pathways. Pharmacokinetics of Mylabris have been conducted to evaluate the efficacy and safety of Mylabris in the clinic, and CTD is stable in plasma, which showed strong penetration and rapid absorption in all tissues, following a one-compartment model with peak concentrations in the liver and kidney of rats observed at 72 h post-administration. Traditional processing methods of Mylabris, such as rice stir-frying and a new targeted delivery system, including mPEG-PLGA micellar encapsulation, metal–organic framework (PPy-CTD@MIL-100@MPCM) nanoparticles, and liposomes, have been developed to enhance the delivery and reduce the systemic toxicity of Mylabris.

However, current research on the pharmacological basis of Mylabris primarily focuses on CTD and its antitumor activity, with a limited understanding of the pharmacological and toxicological profiles of other bioactive constituents of Mylabris. While most toxicity studies of Mylabris remain confined to histopathological assessments, and mainly focus on liver and kidney toxicity, with less research on other toxicities and mechanisms. Pharmacokinetics of Mylabris and its active ingredients mainly focus on blood concentration and tissue distribution, lacking analysis of its metabolic transformation in vivo. Furthermore, the lack of standardized protocols for dose optimization and toxicity relief in clinical applications hinders further clinical applications. Future research directions should focus on exploring other pharmacological activities of CTD, such as insect resistance, antibacterial and antiparasitic activities, as well as strengthening the identification of new active compounds of Mylabris, conducting comprehensive studies on the pharmacological and toxicological mechanisms of Mylabris and other active ingredients, and conducting pharmacokinetics and clinical studies. The exploration of novel delivery systems, detoxification strategies and the development of clinical standardized medication guidelines could enhance the clinical utility of Mylabris, thereby reducing side effects and improving patient outcomes. Furthermore, combining mass spectrometry imaging technology to study the in situ distribution of active ingredients in Mylabris can further clarify its pharmacological action sites. The application of single-cell multi-omics technology offers the potential to accurately identify the pharmacological targets and mechanisms of toxicity accumulation in Mylabris. This approach can enhance the efficacy and reduce the toxicity of Mylabris, thereby advancing the development of clinical translational research.

## Data Availability

Data will be made available on request.

## Abbreviations

TCM: Traditional Chinese medicine
CTD: Cantharidin
CA: Cantharidic acid
SC: Sodium cantharidinate
PPI/2A: Protein phosphatases 1/2A
UPLC-Q-TOF–MS: Ultra-performance liquid chromatography-quadrupole time-of-flight-tandem mass spectrometry
SPME: Solid-phase microextraction
GC–MS: Gas chromatography-mass spectrometry
TP53: Tumor protein 53
DKK3: Dickkopf-3
JNK: C-Jun N-terminal kinase
ERS: Endoplasmic reticulum stress
EphB4: Eph receptor B4
JAK2: Janus kinase 2
STAT3: Signal transducer and activator of transcription 3
PI3K: Phosphatidylinositol-4,5-bisphosphate 3-kinase
PI3K: Phosphatidylinositol-4,5-bisphosphate 3-kinase
Akt: Protein kinase B
PKM2: Pyruvate kinase M2
LC3: Microtubule-associated protein 1 light chain 3
MAPK: Mitogen-activated protein kinase
EGFR: Epidermal growth factor receptor
ERK: Extracellular signal-regulated kinase
PKC: Protein kinase C
CDK1: Cyclin-dependent kinase 1
CCAT1: Colon cancer associated transcript 1
MMP2/9: Matrix metalloproteinases 2 and 9
PTPN1: Protein tyrosine phosphatase non-receptor type 1
AFP: Alpha-fetoprotein
ALT: Alanine aminotransferase
AST: Aspartate aminotransferase
LDH: Lactate dehydrogenase
AMPK: AMP-activated protein kinase
HIF-1: Hypoxia-inducible factor-1
PERK: Protein kinase RNA-like endoplasmic reticulum kinase
CHOP: Enhancer-binding protein homologous protein
NLRP3: Nucleotide-binding oligomerization domain, leucine-rich repeat and pyrin domain-containing 3
TN-T: Troponin T
GL: Glycyrrhizic acid
GA: Glycyrrhetinic acid
FA: Folate
